# Editorial: Botox in Complex Abdominal Wall Surgery

**DOI:** 10.3389/fsurg.2022.900968

**Published:** 2022-04-18

**Authors:** Jose Bueno-Lledo, Nabeel Ibrahim, Martin Zielinski

**Affiliations:** ^1^Unit of Abdominal Wall Surgery, Department of Surgery, La Fe Hospital, Valencia, Spain; ^2^Department of Surgery, Macquarie University, Sydney, NSW, Australia; ^3^Department of Surgery, Mayo Clinic, Rochester, MN, United States

**Keywords:** botox, botulinum toxin type A, abdominal wall, pneumoperitoneum, hernia repair, preoperative techniques, abdominal CT, large ventral hernia

Repair of large ventral hernias (LVH) represents a challenge for the abdominal wall surgeon. These large hernias are usually associated with important parietal reduction that cannot be repaired with a simple technique of fascial closure. Chronic muscle retraction and contracture reduce the volume of the peritoneal cavity and make fascial closure fraught with potential problems such as abdominal compartment syndrome, ventilatory restriction, and an elevated risk of hernia recurrence. Adequate preoperative preparation of patients with LVH is crucial to avoid serious postoperative complications and to assure satisfactory results after hernia repair. For this reason, the use of the botulinum toxin type A (BTA) has emerged as a complementary therapy to facilitate this repair (1).

BTA induces a reversible flaccid paralysis of the lateral abdominal muscle, and has been considered as a “chemical component separation” (2). BTA has been used successfully for delayed abdominal wall reconstruction after damage control laparotomy, open abdomen management and treatment of LVH (3). This neurotoxin has demonstrated to enlarge the abdominal cavity by stretching and flattening of the retracted lateral wall, increasing the abdominal capacity prior to elective hernia repair (Whitehead-Clarke and Windsor). Maximum effect is reached in 2 weeks. It also presents the advantage of continued effect in the late postoperative period, ~6 months during which the operated abdomen adapts to these changes. Ibarra-Hurtado et al. was one of the pioneers in the use of the application of BTA in abdominal wall surgery (4). He initially reported administration of BTA on the lateral abdominal wall muscles to obtain transient flaccid paralysis and decreasing the transverse diameter of the defect.

Another use of BTA allows getting a successful down-staging of the surgical repair in cases of LVH, especially with hernia transverse diameters over 11 cms and elimination of muscular lateral traction (5). These results contribute to minimise disadvantages associated to techniques like component separation technique in terms of the surgical site occurrences, as well as the possibility of tension-free closure, decreasing the length of hospital stay and hernia recurrence rate.

Most groups use ultrasound guidance to locate the injection point of the neurotoxin. Electromyographic guidance is a useful and complementary tool for each BTA injection, apart of the ultrasonography. It may be useful to identify precisely and confirm whether the muscle where the toxin is applied is denervated or fibrotic depending on the history of previous surgeries or the kind of surgical incision, improving its effectiveness (6).

Significant changes in abdominal volumes and hernia transverse diameters in abdominal CT scan with Valsalva manoeuvre have been demonstrated (Figure 1). CT scan is essential in preoperative planning of the LVH and the BTA management. This method has been improved by more accurate radiologic images, which can obtain the length and thickness of the lateral muscles in order to compare the effect of BTA. Tanaka was the first author to provide an objective method for calculating diameters and volumes according CT findings (7). In fact, loss of domain hernia (LODH) has been defined based on these radiologic measurements: a relation hernia sac (HV)/abdominal cavity (ACV) volumes over 20% would cause a traumatic reduction and possible postoperative complications.

**Figure 1:**
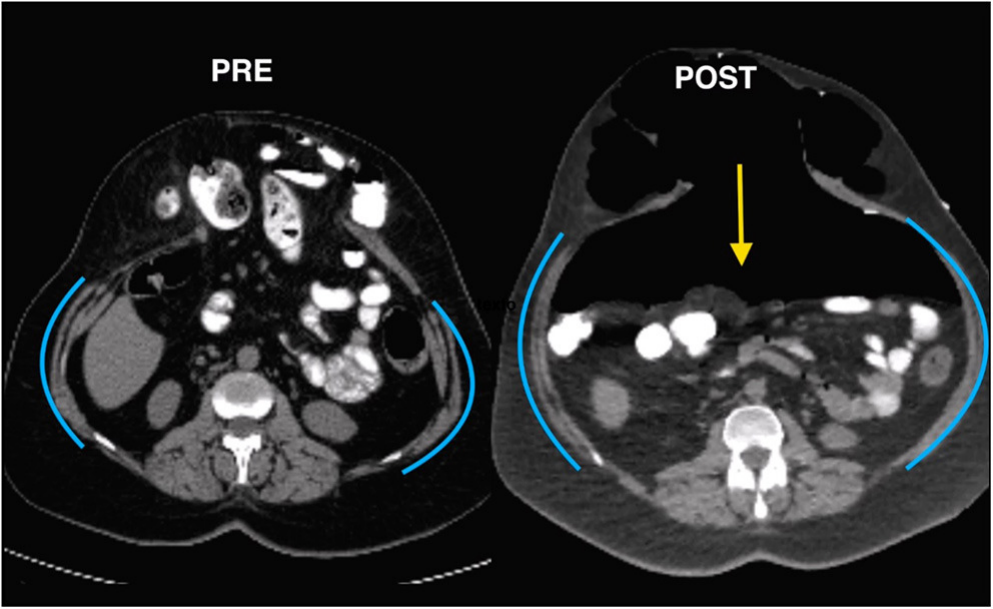
Changes in abdominal volumes and hernia diameters in CT after BTA and PPP.

Preoperative progressive pneumoperitoneum (PPP) was introduced by Goñi Moreno (PMID: 5096685). By this technique, the abdominal cavity can be expanded in order to maintain in place the herniated volume and improve the respiratory adaptation. Use of PPP in the treatment of LVH has been previously reported (8) providing complete repair and reducing complications, such as the abdominal compartment syndrome and the restrictive respiratory disease. Although it is not universally used in most centres, specialized groups have reported good results with this procedure, with acceptable risks (Elstner et al.). There are no clear recommendations regarding the optimal duration, frequency, or right volume of the insufflations. The main drawback of these approaches is that they were not based on an objective factor, and the measurements are neither reliable nor reproducible, so they were hardly standardized.

The combination of both techniques is useful in the management of LVH, to treat diameters and volumes of the hernia as a pre-operative preparation to the surgical repair (Tang et al.). Although more prospective studies comparing both techniques are needed to confirm these results, BTA and PPP significantly reduce the VIH/VAC ratio, which constitutes an important factor in the treatment of LVH (9). Consequently, comparing both preoperative techniques should be the aim of future studies.

## Author Contributions

The main idea of the paper was reported by JB-L. NI and MZ have completed the proof with their support and experience. All authors have collaborated in this manuscript. All authors contributed to the article and approved the submitted version.

## Conflict of Interest

The authors declare that the research was conducted in the absence of any commercial or financial relationships that could be construed as a potential conflict of interest.

## Publisher's Note

All claims expressed in this article are solely those of the authors and do not necessarily represent those of their affiliated organizations, or those of the publisher, the editors and the reviewers. Any product that may be evaluated in this article, or claim that may be made by its manufacturer, is not guaranteed or endorsed by the publisher.
